# Small Intestinal Ischemia with Pneumatosis in a Young Adult: What Could Be the Cause?

**DOI:** 10.1155/2013/462985

**Published:** 2013-02-05

**Authors:** Amara Jyothi Nidimusili, Jenna Mennella, Khaldoon Shaheen

**Affiliations:** ^1^Department of Internal Medicine, St. Joseph's Regional Medical Center, Paterson, NJ, USA; ^2^Department of Hospital Medicine, Institute of Medicine, Cleveland Clinic, Cleveland, OH 44195, USA

## Abstract

This case highlights one of the infrequent complications of a commonly abused substance. A particular high index of suspicion of ischemic bowel is associated with cocaine abuse and should be included in the differential diagnosis of any young adult or middle-aged patient with abdominal pain and/or bloody diarrhea, particularly in the absence of other predisposing factors. To our knowledge, we report a rare case of ischemic small bowel associated with gangrene and pneumatosis intestinalis due to cocaine abuse.

## 1. Introduction

This case highlights one of the infrequent complications of a commonly abused substance. A particular high index of suspicion of ischemic bowel is associated with cocaine abuse and should be included in the differential diagnosis of any young adult or middle-aged patient with abdominal pain and/or bloody diarrhea, particularly in the absence of other predisposing factors. Up to our knowledge, we report a rare case of ischemic small bowel associated with gangrene and pneumatosis intestinalis due to cocaine abuse.

## 2. The Case

**Figure 1:**
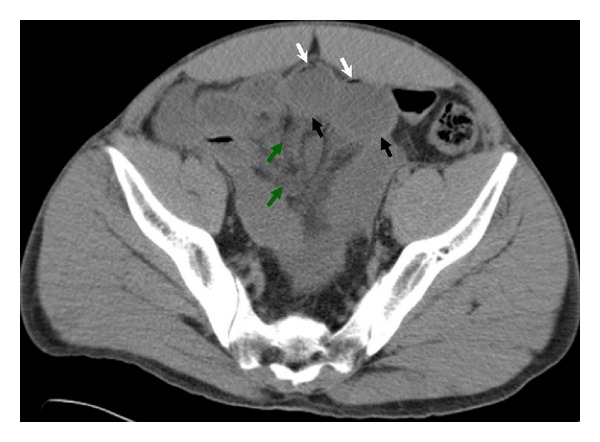
Computed tomography of the abdomen reveals multiple loops of mid and distal small bowel were dilated (black arrows) with pneumatosis intestinalis. The white arrows mark areas of air in the small bowel wall. The surrounding mesentery is edematous as marked with the green arrows.

**Figure 2:**
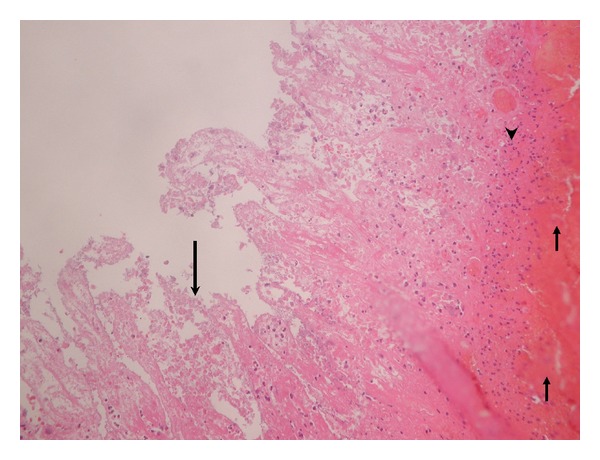
Histopathology signs of ischemic intestine of the resected small bowel show diffuse denuded mucosal surface (long arrow) with hemorrhagic infarction changes (short arrows), and superficial neutrophilic inflammation and fibrin deposition are seen within the lamina propria (arrow head). Hematoxylin-eosin stain, ×200.

A 36-year-old Caucasian man presented to the emergency room with one-day history of acute onset right lower quadrant abdominal pain with nausea and vomiting. He denied any diarrhea, constipation, rectal bleed, fever, chills, food intolerance, or change in appetite. His medical and surgical history was unremarkable except for an appendectomy 10 years ago; he had never had a colonoscopy. His social history was significant for smoking (20-pack years) and occasional alcohol use. He denied the use of illicit drugs. On presentation, the patient was afebrile; other vital signs were within normal limits. He appeared well developed, well nourished, and in no acute distress. His abdomen was soft, with increased tenderness to palpation in the right lower quadrant with no rebound tenderness or palpable masses. The remainder of the physical examination, including a rectal examination, was unremarkable, and a test for fecal occult blood was negative. Initial blood workup revealed a slightly elevated white blood cell count of 12,500 cells/mL (4,500–11,000 cells/mL) with a normal differential. Serum calcium, kidney and liver function, and urinalysis results were normal. Computed tomography (CT scan) of abdomen and pelvis revealed only a 2 mm nonobstructing right lower renal stone with no hydronephrosis. Over the next 24 hours, however, the patient's abdominal pain worsened. He was in obvious distress. Vital signs included tachypnea in the 30 s and a sinus tachycardia to the 130 s. The remainder of the physical exam revealed a diffusely tender, tympanitic, and distended abdomen with guarding and rebound tenderness. Laboratory analyses revealed an elevated white blood cell count of 16,900 cells/mL and an elevated lactic acid of 5.5 mmol/L (0.5–2.2 mmol/L). Repeated CT scan of the abdomen and pelvis showed multiple loops of mid and distal small bowel were dilated with pneumatosis intestinalis (Figure 1). Following fluid resuscitation and antibiotic administration, the patient was taken for exploratory laparotomy. Intraoperatively, he was found to have foul smelling, turbid loop of gangrenous bowel with fibrinous exudate. Approximately 20 cm of frankly necrotic jejunum and ileum were resected. There were no internal hernia and no adhesive bands found. The patient was then taken back to the intensive care unit for further resuscitation. A temporary abdominal vacuum device was placed. Pathology revealed evidence of transmural ischemia (Figure 2). Reexploration on postoperative day 1 showed no further ischemia. The postoperative recovery was uneventful. The youth of the patient and his unexplained colonic ischemia placed coagulation disorders and drug abuse at the top of the differential diagnosis; therefore, thrombophilia and toxicology screens were performed. The thrombophilia screen was negative, but the urine toxicology screen was positive for marijuana and cocaine. A thorough interview eventually revealed that the patient had engaged in recreational cocaine and Molly (3,4-methylenedioxy-N-methylamphetamine—MDMA) use a few days before presentation and confirmed the diagnosis of cocaine-induced intestinal ischemia.

## 3. Discussion

Intestinal Ischemia is more common in elderly patients but also is seen in younger patients [1]. The causes can be broadly divided into occlusive and nonocclusive entities. The most common mechanisms are nonocclusive: hypotension caused by sepsis or impaired left ventricular function and hypovolemia caused by dehydration or hemorrhage. Other causes include occlusive atherosclerotic plaques, thromboses, thromboemboli, dissections, mechanical obstructions, blood dyscrasias, major cardiac and postaortic reconstruction surgeries, vasculitis, amyloidosis, coagulation disorders, long-distance running, and drugs [1].

Although the complications of cocaine abuse are generally more likely to be cardiovascular or respiratory in nature than gastrointestinal [2], cocaine use can affect the entire gastrointestinal tract. Complications of the upper gastrointestinal tract (prepyloric or duodenal perforation) are more common than complications of the lower gastrointestinal tract (bowel ischemia, gangrene, or perforation) [2]. Although intestinal ischemia in cocaine users has been described in the literature, the distal ileum is the most commonly affected small bowel segment [3]. Bowel injury occurs via a pathophysiologic mechanism in which cocaine blocks the reuptake of norepinephrine, leading to arterial vasospasm or vasoconstriction and subsequent intestinal ischemia with mucosal and transmural necrosis [2]. Cocaine also has a direct toxicity on gut mucosa which further contributes to intestinal ischemia [1].

Ischemic injury occurs most often in the areas of the intestine that are more vulnerable to a low-flow state, such as the watershed areas (the splenic flexure and rectosigmoid junction), which have limited collateral networks. Although any area of the intestine can be affected, approximately 75% of cases involve the left colon and approximately 25% of these involve the splenic flexure. In recent series, the right colon was found to be involved in 12% to 47% of cases [1].

In a literature search, we found approximately 30 reported cases of cocaine-induced bowel ischemia. Most patients were in their third or fourth decade of life [4], and the oldest patient was a 50-year-old man [3]. Cocaine-induced bowel ischemia was first described in 1985, when the recreational use of cocaine was dramatically increasing. Although no method of cocaine administration is safe, the nasal route is probably least likely to cause bowel ischemia [3]. The interval between drug ingestion and the onset of symptoms varies from 1 hour to 2 days [2, 3]. After the intake of the drug, abusers commonly develop abdominal pain and tenderness, and bloody diarrhea may occur [2]. Most cases reported in the literature have involved ischemia of the small, rather than the large, bowel [3].

In the present case of ischemic bowel in a 36-year-old man, the cause was the recreational cocaine use. Our patient had no history of other known predisposing factors for bowel ischemia, although his cigarette smoking might have been a contributory factor. Cigarette smoking is common among those who use cocaine, and the vasoconstrictive effects of cocaine and nicotine, which are probably additive [3, 5], also might affect the mesenteric vessels. We conclude that ischemic bowel associated with cocaine use is a rare complication that should be included in the differential diagnosis of any young adult or middle-aged patient with abdominal pain and bloody diarrhea, particularly in the absence of other predisposing factors [5].

Although computed tomography findings can suggest ischemic bowel or demonstrate its extent, colonoscopy, which establishes a definitive diagnosis, is the diagnostic procedure of choice when the large bowel is involved [1]. In cases of small bowel involvement, catastrophic presentation is more encountered, and generally exploratory laparatomy is required. In the majority of patients with bowel ischemia, angiography cannot identify a specific lesion occluding a major artery to the intestine; instead, it often reveals patent major visceral vessels, probably because most vessel lesions causing ischemic bowel are peripheral or the cause is nonocclusive. Therefore, although angiography is an option for most patients with bowel ischemia, it is generally not useful in patients with cocaine-induced bowel ischemia [1].

The clinical course of ischemic bowel varies. In the majority of patients with ischemic colitis (85%), the ischemia involves the mucosa and submucosa, and the prognosis is good; the ischemia is transient and self-limited and heals uneventfully with conservative management. A minority with the chronic subtype may have sequelae of persistent segmental colitis or colonic strictures, some of which might require surgery. In the most unfortunate patients, delayed diagnosis leads to acute fulminant ischemia with transmural infarction that may progress to necrosis and death; these patients deteriorate with conservative management, and the majority require surgery [1]. Wattoo et al. reviewed the literature and reported that about two thirds of patients presenting with cocaine-induced intestinal ischemia survived with a majority requiring surgery. If peritonitis, gastrointestinal tract perforation, or marked leukocytosis is evident, exploratory laparotomy must be considered [5].

## 4. Conclusion

Cocaine-induced bowel ischemia is a rare reported presentation of cocaine abuse but should be included in the differential diagnosis of young or middle-aged risky patients with abdominal pain and/or bloody diarrhea. Clinicians should recognize the importance of drugs screening in young adults with visceral ischemia.
