# Erratum to “Small Intestinal Ischemia with Pneumatosis in a Young Adult: What Could Be the Cause?”

**DOI:** 10.1155/2013/846037

**Published:** 2013-05-07

**Authors:** Amara Jyothi Nidimusili, Jenna Mennella, Khaldoon Shaheen

**Affiliations:** ^1^Department of Medicine, Trinitas Regional Medical Center, Seton Hall University Health Sciences, Elizabeth, NJ 07202, USA; ^2^Department of Hospital Medicine, Institute of Medicine, Cleveland Clinic, Cleveland, OH 44195, USA

The affiliation of Dr. Nidimusili and Dr. Mennella is corrected to be “Department of Medicine, Trinitas Regional Medical Center, Seton Hall University Health Sciences, Elizabeth, NJ 07202, USA.”

